# SB202190-Induced Cell Type-Specific Vacuole Formation and Defective Autophagy Do Not Depend on p38 MAP Kinase Inhibition

**DOI:** 10.1371/journal.pone.0023054

**Published:** 2011-08-10

**Authors:** Manoj B. Menon, Alexey Kotlyarov, Matthias Gaestel

**Affiliations:** Institute of Biochemistry, Hannover Medical School, Hannover, Germany; University Medical Center Groningen, The Netherlands

## Abstract

SB202190, a widely used inhibitor of p38 MAPKα and β, was recently described to induce autophagic vacuoles and cell death in colon and ovarian cancer cells lines and, therefore, this effect was supposed to be specific for transformed cells and to open therapeutic options. Here, we demonstrate that SB202190 and the structurally related inhibitor SB203580 induce pro-autophagic gene expression and vacuole formation in various cancer and non-cancer cell lines of human, rat, mouse and hamster origin. This effect seems to induce defective autophagy leading to the accumulation of acidic vacuoles, p62 protein and lipid conjugated LC3. Using further p38 inhibitors we show that p38 MAPK inhibition is not sufficient for the autophagic response. In line with these results, expression of a SB202190-resistant mutant of p38α, which significantly increases activity of the p38 pathway under inhibitory conditions, does not block SB202190-dependent vacuole formation, indicating that lack of p38α activity is not necessary for this effect. Obviously, the induction of autophagic vacuole formation by SB203580 and SB202190 is due to off-target effects of these inhibitors on post-translational protein modifications, such as phosphorylation of the MAPKs ERK1/2 and JNK1/2, ribosomal protein S6, and PKB/Akt. Interestingly, the PI3K-inhibitor wortmannin induces transient vacuole formation indicating that the PI3K-PKB/Akt-mTOR pathway is essential for preventing autophagy and that cross-inhibition of this pathway by SB202190 could be the reason for the early part of the effect observed.

## Introduction

Small molecule protein kinase inhibitors are largely being developed for the treatment of a variety of human diseases [1], [2]. p38 MAPK has been identified as a potential target of such small molecules for the treatment of cancer and inflammation [3]. SB203580 and SB202190 are the most widely used inhibitors of the p38 MAPK pathway. The gate keeper threonine (T) 106 in the ATP-binding groove of p38α was shown to be the major determinant for the specificity of this class of compounds. The majority of the known protein kinases carry a bulky residue at T106 equivalent position, which prevents binding of SB203580 and SB202190 [4], [5]. Although the SB compounds were thought to specifically inhibit the α and β isoforms of p38 MAPK leading to suppression of inflammatory gene expression, later studies identified several further protein kinase targets of these compounds including GAK, GSK3β, RICK (RIP2), Casein kinase I, Type-II TGF receptor, LCK, CRAF (Raf-1), BRAF and PDK1 [5], [6], [7]. In addition, at higher concentrations SB compounds were shown to have inhibitory effects on several non-protein kinase targets, such as hepatic cytochrome P450 enzymes [8], cyclooxygenases and thromboxane synthase [9].

SB202190 was shown to induce autophagic vacuoles and cell death in a colon-cancer specific manner [10]. This observation was recently extended to ovarian cancer cells [11] and suggested an important role of p38 MAPK and serious therapeutic potential for SB202190 in colon cancer treatment. The observed macro-autophagy is an evolutionarily conserved process highly active during differentiation and development, consisting of the sequestration of cytoplasmic proteins and organelles into autophagosome, with subsequent degradation in the autophagolysosomes [12], [13]. While the major regulator of autophagy is the mTOR pathway, which regulates the rate of autophagy in response to nutrient availability [14], recent studies have demonstrated the importance of p38 and ERK1/2 MAPK signaling in the formation and maturation of autophagic vacuoles in response to starvation and several other chemical stresses [15], [16], [17], [18]. Furthermore, inhibition of p38 MAPK by SB202190 was demonstrated to induce transcriptional reprogramming which involves a shift from HIF-1α-dependent to Foxo-3A-dependent pro-autophagic gene expression leading to type-II programmed cell death [19]. The vacuoles induced by SB compounds were unusually large and, hence, reminiscent of blockade of autophagic clearance rather than an efficient autophagic flux. Here, we analyzed the effect of SB202190 with the aim to further characterize the nature of this cell-type specific vacuolation response and the role of p38 MAPK in autophagy. Unexpectedly, our results indicate that neither inhibition of p38 MAPK nor the gene expression changes are necessary for the SB202190-mediated autophagic response, which seems to be cell type- but not cancer-specific. Instead, SB202190 interferes with various signaling pathway including PI3K/Akt/mTOR signaling, which could be responsible for the autophagic response observed.

## Results

### SB202190 induces cytoplasmic vacuoles in a cell type-specific manner in transformed and non-transformed cells

**Figure 1:**
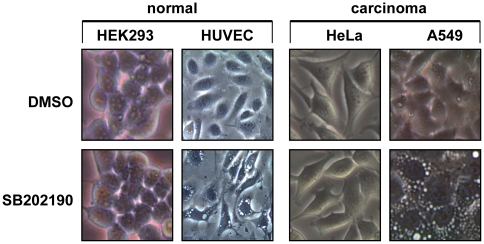
Cell type-specific induction of vacuoles by SB202190. Indicated cells were treated with 5 µM SB202190 or solvent control (DMSO) for 12 h and phase contrast images taken using a 20× objective a and 30× digital camera magnification factor.

SB202190 is the most widely used p38 MAPK inhibitor. Treatment with 5 µM SB202190 reproducibly induced vacuole formation in various cell lines while it does not in other cells (Figure 1, Table 1). The induction of vacuole formation does not correlate with the transformation status of the cells, since it is detected also in non-transformed cell lines (BHK21, IEC6, RGM1) as well as primary cells (HUVEC, MEFs, hMSC) and could not be detected in some of the transformed cells tested (HeLa, Sh-SY5Y, WM1617, WM793). Vacuoles were clearly visible in most of the sensitive cell lines after approximately 2 h of SB202190 treatment.

**Table 1:** Cell type-specific vacuole formation induced by SB202190.

No	Cell line	Species	Cell type	Vacuoles
1	**AGS**	Human	gastric adenocarcinoma	**+**
2	**A549**	Human	lung carcinoma	**+**
3	**BHK21**	Hamster	adult kidney fibroblast	**+**
4	**C2C12**	Mouse	Myoblast	**−**
5	**Caco-2-Bbe**	Human	colorectal adenocarcinoma	**+**
6	**HEK293T**	Human	embryonic kidney	**−**
7	**HeLa**	Human	cervical adenocarcinoma	**−**
8	**hMSC**	Human	mesenchymal stem cells	**+**
9	**HT29**	Human	colorectal adenocarcinoma	**+**
10	**HUVEC**	Human	endothelial cells	**+**
11	**IEC6**	Rat	small intestinal epithelium	**+**
12	**L929**	Mouse	Fibrosarcoma	**+**
13	**MEF**	Mouse	embryonic fibroblast	**+**
14	**NIH 3T3**	Mouse	embryonic fibroblast	**−**
15	**RGM1**	Rat	gastric epithelium	**+**
16	**Sh-SY5Y**	Human	Neuroblastoma	**−**
17	**WM1617**	Human	Melanoma	**−**
18	**WM793**	Human	Melanoma	**−**

### SB202190 induces accumulation of enlarged autophagolysosomes in HT29 cells

**Figure 2:**
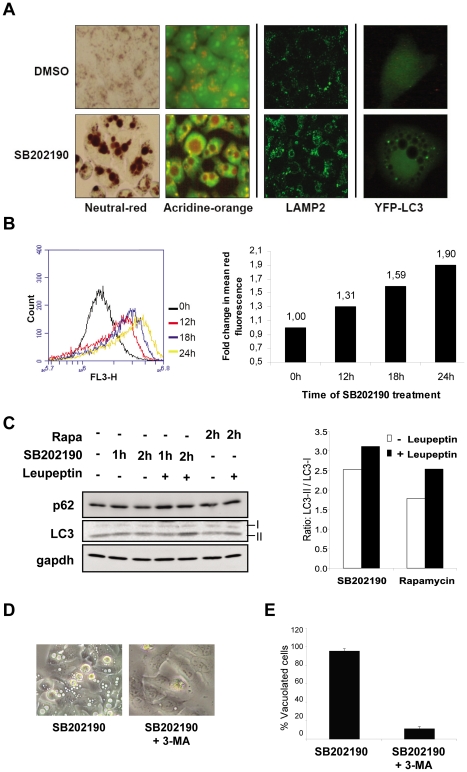
Characterization of SB202190-induced vacuoles in HT29 cells. (**A**) HT29 human colorectal adenocarcinoma cells were treated with 5 µM SB202190 or solvent control (DMSO) for 24 hours and living cells were stained with neutral red or acridine orange (AO) (left panel). Neutral red accumulated in the acidic vacuoles within 10 minutes. AO stained the nucleus and cytoplasm green with the acidic vacuoles visible as orange-yellow structures. Cells treated with DMSO or 5 µM SB202190 for 6 hours were stained for LAMP2 after para-formaldehyde fixation (middle panel). HT29 cells were transiently transfected with YFP-LC3 construct, 24 hours post transfection treated with 5 µM SB202190 for additional 24 hours or left untreated and analyzed for YFP fluorescence (right panel). LC3 dots are detected close to the large non-stained vacuoles. (**B**) HT29 cells were treated with 5 µM SB202190 for indicated times, stained with AO and red fluorescence (FL3 channel) was quantified by FACS. The change of mean fluorescence was calculated and shown in the right panel. (**C**) Cells in complete growth medium were treated with 5 µM SB202190 or 50 nM rapamycin in the presence or absence of 200 µg/mL leupeptin for indicated time points. Ratios of the band intensities for LC3-II to LC3-I is plotted in the right panel (**D**) HT29 cells were treated with SB202190 (5 µM) in the presence or absence of 10 mM 3-methyl adenine for 12 h. (**E**) Percentage of vacuolated cells were calculated from 3 independent images and plotted in the right panel.

Previous studies have reported colon cancer-specific induction of vacuoles and autophagic cell death by SB202190. Since this effect was originally analyzed and characterized for its autophagic key features in detail in HT29 human colon cancer cells [10], we continued to analyze the SB202190 effect in this cell line, too. Autophagy is characterized by the sequestration of cytoplasmic contents by membranous autophagosomes and degradation by the lysosomal pathway. The autophagosomes are usually ultrastructures often visualized by electron microscopy and hence the very large vacuoles induced by SB202190 probably represent artifacts of an immature autophagic response. As shown by acridine orange (AO) and neutral red staining of SB202190-treated HT29 cells, the vacuoles were highly acidic (Figure 2A, left panel). SB202190-induced vacuoles were also positive for the presence of the lysosomal membrane protein LAMP2 (Figure 2A, middle panel). Induction of autophagy is characterized by the conversion of microtubule-associated protein 1 light chain 3 (LC3) to a lipid conjugated form, LC3-II, and its recruitment to autophagic vacuoles forming a distinct punctuate staining pattern [20]. After SB202190 treatment, transfected YFP-tagged LC3 showed punctate structures close to the enlarged vacuoles, but did not co-localize with the vacuoles (Figure 2A, right panel). This is similar to the abnormal autophagolysosomes observed in sertoli cells upon lindane treatment [15]. Since these vacuoles are LAMP2 positive, acidic and hence of lysosomal origin, we quantified the SB202190-induced vacuolation response by assessing the acidic vacuolar content by acridine orange (AO) staining. For that reason, we stained SB202190-treated HT29 cells and measured the red fluorescence of AO by FACS. A significant time-dependent up regulation of red fluorescence of AO in response to SB202190 treatment could clearly be detected (Figure 2B).

We also analyzed endogenous LC3 in HT29 cells by western blotting. There is a significant level of basal LC3-II in HT29 cells, which is not further increased by 2 h of SB202190 treatment. However, SB202190 treatment together with lysosomal protease inhibitor led to further accumulation of lipid conjugated LC3, suggesting the presence of active autophagic flux (Figure 2C). Other markers for autophagic response, such as Beclin-1 and ATG12-ATG5, showed no significant changes (data not shown). Interestingly, the treatment of HT29 cells with rapamycin, an inhibitor of mTOR and a commonly used autophagy inducer, also showed similar autophagic turnover, as monitored by the LC3-II/LC3-I ratio in the presence and absence of protease inhibitor (Figure 2C). It was interesting to note that rapamycin did not induce the formation of vacuoles (data not shown). Consistent with previous studies, treatment with 3 methyl adenine (3MA), a class III PI3 kinase inhibitor and inhibitor of autophagy could suppress SB202190-induced vacuole formation (Figure 2D, E). Additionally, chloroquine, an inhibitor of lysosomal acidification, and wortmannin, a broad spectrum PI3 kinase inhibitor, also prevented vacuole formation (Figure S1). Thus, PI3 kinase activity, LC3 lipid conjugation and lysosomal acidification seem prerequisites for the vacuolation response.

### SB202190 treatment leads to non-productive autophagy and accumulation of autophagic substrates

**Figure 3:**
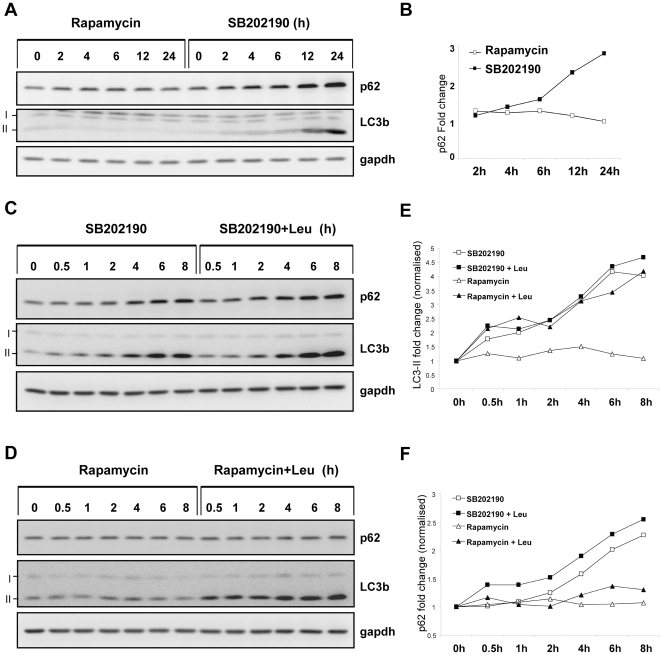
Differential effects of SB202190 and rapamycin on autophagic proteolysis. (**A**) Cells were treated for indicated time periods with 50 nM rapamycin or 5 µM SB202190 and lysates were analyzed for LC3b and p62 levels by western blotting. (**B**) p62 band intensities from panel A was quantified using Image J software, normalized to gapdh and plotted. (**C**) HT29 cells were treated for indicated time periods with SB202190 (10 µM) in the presence or absence of 200 µg/mL leupeptin and autophagy substrates analyzed by western blot (**D**) Cells were treated with rapamycin (100 nM) alone or in combination with leupeptin and analyzed (**E**) LC3-II band intensities from panel C and D were quantified as in panel B (**F**) p62 band intensities from panel C and D were quantified as described in panel B.

Involvement of p38 MAPK in autophagy regulation is complex and different studies have suggested opposing roles for the pathway in starvation induced autophagy. Hence, we hypothesized that the differences between rapamycin and SB202190 could be due to a late effect of p38 inhibition which blocks autophagolysosome resolution, and hence accumulation of these acidic vacuoles. To prove this hypothesis we monitored the levels of p62, a substrate protein preferably degraded by the autophagic pathway in SB202190 and rapamycin treated cells in parallel [21]. Rapamycin did not drastically affect endogenous p62 protein levels in HT29 cells, while SB202190 strongly up regulated p62 levels in HT29 cells at later time points (Figure 3A, B). Lipid conjugated LC3 (LC3 II) also accumulated at 12 and 24 h of SB202190 treatment, supporting a model of enhanced lipid conjugation and reduced flux by lysosomal degradation (Figure 3A). We further verified this by analyzing the autophagic turnover of LC3-II and p62 in the presence or absence of leupeptin, a lysosomal protease inhibitor. When HT29 cells were treated with SB202190, a time-dependent accumulation of p62 and LC3-II independent of the presence or absence of leupeptin was observed (Figure 3C, E & F). In contrast, rapamycin treatment alone did not show significant effects on the levels of LC3-II or p62. Rapamycin in combination with leupeptin led to strong LC3 lipid conjugation and a small, but significant up regulation of p62 (Figure 3D, E & F). This observation conclusively demonstrates that in the identical cellular system, the turnover of autophagy substrates are differentially affected by rapamycin and SB202190. Rapamycin induces active autophagic proteolysis sensitive to leupeptin treatment, while SB202190 induces non-productive autophagy, accumulation of p62 and LC3-II. In addition nutrient starvation-induced autophagic proteolysis was also significantly suppressed by SB202190 treatment (Figure S2).

### SB202190-induced *de-novo* gene expression is not necessary for vacuolation in HT29 cells

**Figure 4:**
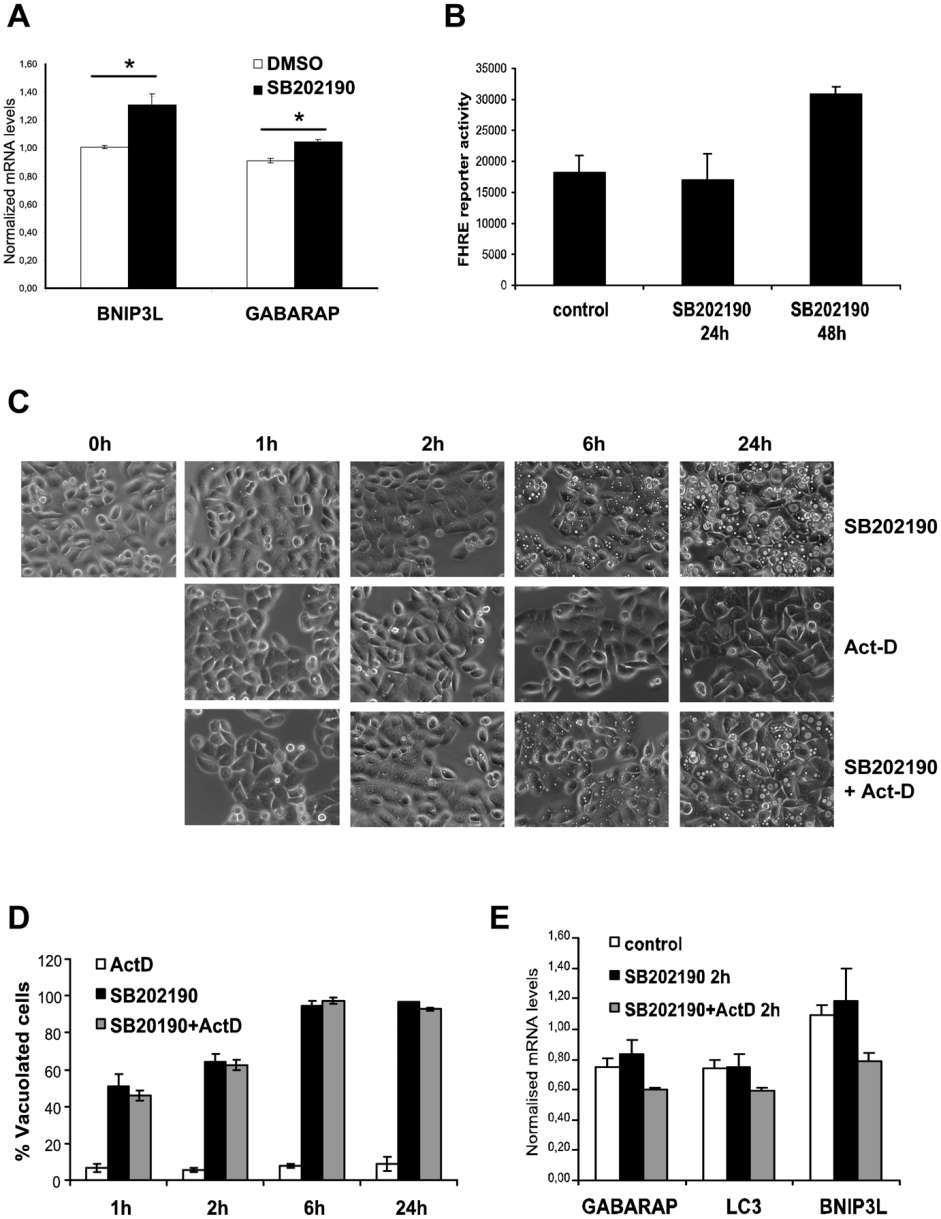
SB202190-induced transcriptional changes are not necessary for vacuole formation in HT29 cells. (**A**) HT29 cells were treated in the presence of DMSO or SB202190 (5 µM) for 24 hours and the levels of BNIP3L and GABARAP mRNAs were analyzed by RT- PCR. The relative mRNA levels were normalized to actin mRNA and plotted (* denotes p-value<0.04). (**B**) HT29 cells stably transfected with FHRE-luc reporter were treated with 10 µM SB202190 for indicated time points and luciferase activity measured and normalized to protein content. (**C**) Cells seeded in 12 well plates were treated with Act-D (5 µg/mL), SB202190 (5 µM) or both together and phase contrast images were taken at indicated time points (original magnification 600×). (**D**) Percentage of vacuolated cells was calculated using Image J software. Percentage vacuolated-cells plotted is the average of three measurements from three independent images. (**E**) HT29 cells treated as indicated were analyzed by RT- PCR for the expression of GABARAP, BNIP3L and LC3.

SB202190-induced autophagic response and vacuole accumulation was previously reported to involve a transcriptional reprogramming leading to up regulation of pro-autophagic genes [19], a role which is not established at the moment [21]. Therefore, we decided to characterize the influence of changes in gene expression in SB202190-induced vacuole formation. 24 hours of SB202190 treatment led to a small, but consistent increase in the expression of GABARAP, a homolog of LC3 (Atg8), and of BNIP3L, a protein involved in autophagy and apoptosis (Figure 4A). This transcriptional up regulation of GABARAP and related genes were reported to be due to Foxo3A activation [19], [22]. To monitor transcriptional activity of Foxo, we generated stable Fork head response element reporter (FHRE-luc)-integrated HT29 cells. Interestingly, SB202190 induced FHRE-luc reporter activity was observed only after 48 h of SB202190 treatment (Figure 4B). This raised the question whether SB202190-induced transcriptional response is the direct reason for or a downstream effect of the vacuolation response. To determine whether SB202190-induced vacuole formation is dependent on the up regulation of autophagy related transcripts, we treated the cells with the transcriptional inhibitor actinomycin-D (Act-D). Treatment with Act-D had no effect on the kinetics of SB202190-induced vacuole formation in HT29 cells, challenging the role of *de novo* gene expression in the vacuolation response (Figure 4C, D). Analysis of GABARAP, LC3 and BNIP3L expression by RT-PCR showed very small induction after 2 h of SB202190 treatment which was effectively suppressed by Act-D (Figure 4E). Thus, our results demonstrate that SB202190-induced transcriptional reprogramming cannot be the reason for the primary effect of vacuole formation but could be a downstream effect of accumulated vacuoles. Insignificant up regulation of the GABARAP related transcripts at 2 h time point and very late induction of the FHRE-luc reporter supports this model.

### p38 MAPK inhibition is not sufficient for SB202190-induced vacuole formation and LC3-II accumulation

**Figure 5:**
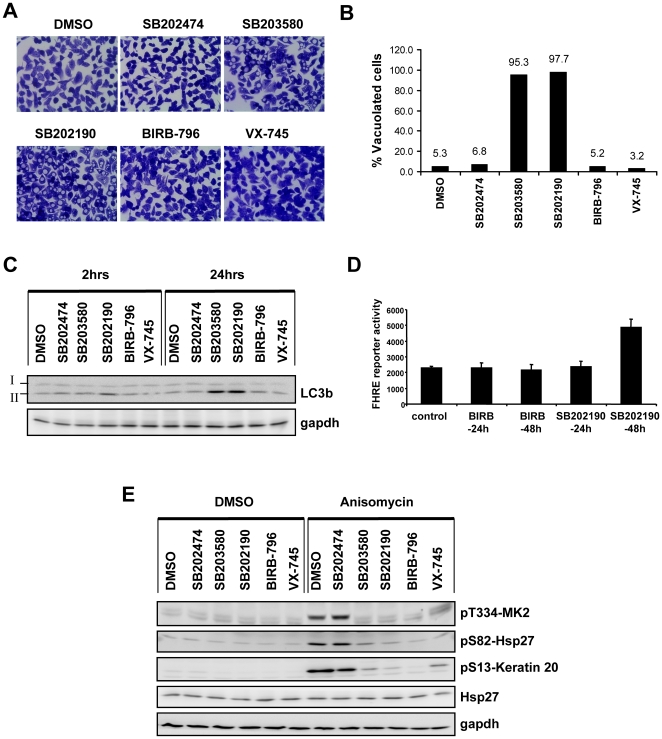
p38 MAPK inhibition is neither sufficient for induction of autophagy nor for FHRE reporter activation. (**A**) HT29 cells were treated for 24 hours with SB203580 (5 µM), SB202190 (5 µM), SB202474 (5 µM), BIRB-796 (1 µM), VX- 745 (2 µM) and DMSO control. Cells were fixed in 95% ethanol and stained with crystal violet (original magnification 600×). (**B**) The percentage of vacuolated cells from three independent images was calculated and the mean values are shown. (**C**) Cells treated with the set of inhibitors as indicated were analyzed by Western blotting for the autophagic marker – LC3b. LC3-I and its lipid conjugated form- LC3-II are indicated. Gapdh was used as loading control. (**D**) HT29 cells stably transfected with FHRE-luc reporter were treated with 10 µM SB202190 or 1 µM BIRB-796 for indicated time points and luciferase activity measured and normalized to protein content. (**E**) Cells were treated with the respective inhibitors or DMSO for 1 hour as indicated, followed by 30 minutes treatment with anisomycin (10 µg/ml) or DMSO. For confirming p38 inhibition, the phosphorylations of MK2, keratin 20 and Hsp27 were monitored by Western blotting. Total Hsp27 and gapdh were used as loading controls.

We then analyzed the vacuolation response in HT29 cells using further p38 MAPK inhibitors (SB203580, BIRB-796 and VX-745) as well as SB202474, an inactive analogue of the SB compounds, as negative control. While SB202190 and SB203580 induce vacuoles, the two other potent inhibitors of p38 MAPK, BIRB-796 and VX-745, unexpectedly failed to induce vacuoles in HT29 cells (Figure 5A, B). Vacuole formation in SB202190- and SB203580-treated cells correlated with the late stage up regulation of LC3-II, which is not up-regulated in response to BIRB-796 and VX-745 (Figure 5C). We monitored FHRE-luc reporter activity between SB202190 and BIRB-796 treated HT29 cells and detected enhanced reporter activity only in response to SB202190 treatment (Figure 5D). Regardless of the ability to induce vacuoles, all inhibitors tested efficiently blocked p38 MAPK α and β activity in these cells as shown by the reduction of phosphorylation of the p38 MAPK substrate MK2 at threonine T334 and its downstream targets Hsp27 (serine S82) and keratin 20 (serine S13) (Figure 5E). We also analyzed the effect of siRNA mediated depletion of p38α on vacuole formation. Even though the siRNA transfection procedure marginally increased the percentage of vacuolated cells, the effect of p38α down regulation was not statistically significant (Figure S3),

### SB202190-induced acidic vacuolation response in HT29 cells proceeds in the presence of p38 MAPK activity

**Figure 6:**
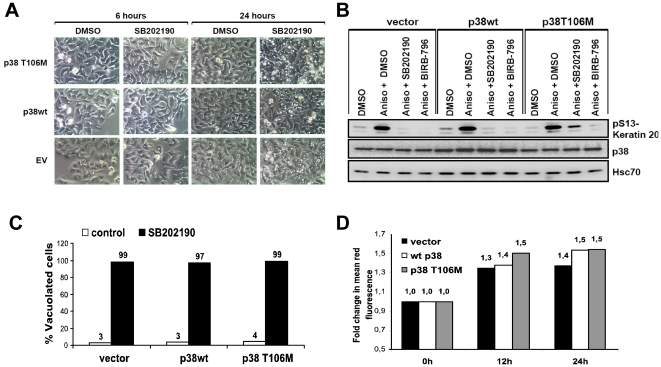
Inhibition of p38 MAPKα is not necessary for SB202190-induced acidic vacuole formation. (**A**) HT29 cells stably transduced with empty vector or vectors expressing wild type p38α or mutant p38α T106M were treated as indicated. Phase contrast images were taken at indicated time points (original magnification 600×) (**B**) p38α-, p38α T106M- or empty vector-transduced cells were treated with 5 µM SB202190 or DMSO as indicated and anisomycin-induced p38 activity monitored by the phosphorylation of keratin 20, a bonafide substrate of MK2. p38 and Hsc70 expression are shown as controls. (**C**) Percentage of vacuolated cells after 6 hours of SB202190 treatment were calculated and plotted. (**D**) Stably transduced cells were treated for indicated times with 5 µM SB202190, stained with AO and analyzed by FACS as described in Figure 2F.

The above experiments demonstrated that inhibition of p38 MAPK alone is not sufficient to explain the massive vacuole accumulation observed in HT29 cells. However, several studies from different genetic models have shown a role for p38 MAPK in the regulation of autophagy [17], [18], [23] and a recent study has proposed that p38 MAPK *α* inhibits autophagy by interfering with the intracellular trafficking of Atg9 [18]. p38 and ERK MAP kinases were shown to coordinately regulate autophagosome maturation [15], [24]. Additionally, MK2, a bonafide substrate of p38 MAPK, was identified as a negative regulator of basal autophagic response in a recent siRNA screen [25]. Since p38 MAPK signaling is therefore important for the regulation of autophagy, we then asked the question whether lack of p38 MAPK activity is necessary for SB-induced autophagolysosome formation, at least. We addressed this question by using the SB-resistant mutant p38 MAPKα-T106M, which fails to bind to and which is not inhibited by SB203580 or SB202190, but otherwise displays normal activation, inhibition by BIRB-796 and activity towards substrates [26], [27]. HT29 cells stably transduced with p38 MAPKα-T106M displayed significantly enhanced anisomycin-induced activity in the presence of SB202190 when compared to cells transduced with wild type p38 MAPK or control vector, as monitored by activation of the MK2-bonafide substrate keratin 20 at serine S13 (Figure 6B) [28]. In contrast to this difference in p38 MAPK activity, there were no significant differences observed between these cells in SB202190-induced vacuole formation (Figure 6A, C). Similar results were obtained for SB203580-induced vacuolization (data not shown). The increase in acidic vacuolar content quantified by AO staining and FACS scan did not show any significant difference between these cell lines, even though there were minor differences in the kinetics of vacuole acidification (Figure 6D). This conclusively shows that down regulation of p38 MAPK activity is not critical for SB-induced accumulation of defective autolysosomes.

### SB202190-specific signalling events in HT29 cells

**Figure 7:**
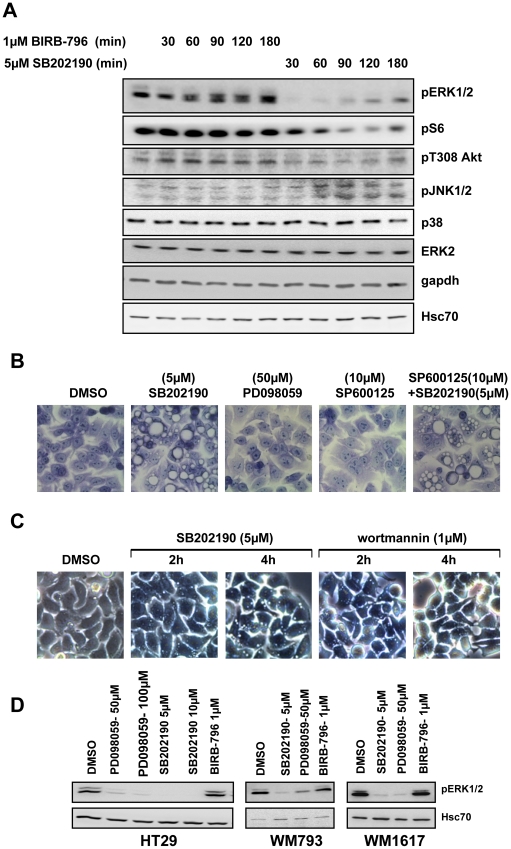
Modulation of different signaling pathways by SB202190 in HT29 cells and analysis of their role in vacuolation response. (**A**) HT29 cells were treated with BIRB-796 (1 µM) or SB202190 (5 µM) for indicated times and analyzed by Western blotting with the indicated antibodies. Hsc70 and gapdh were used as loading controls. (**B**) Cells were treated for 12 h with the indicated inhibitors or solvent control, fixed in 95% ethanol and stained with crystal violet (original magnification 600×). (**C**) Cells were treated with SB202190 or wortmannin and live cell images (original magnification 600×) were acquired at indicated time points. (**D**) HT29, WM1617 and WM793 maintained in complete growth medium were treated with indicated concentrations of SB202190, PD098059 or BIRB-796 for 90 minutes. Lysates were analyzed for phosphorylation of ERK1/2. Hsc70 was used as loading control.

Since the primary effect of SB202190 on vacuolization is independent of *de novo* gene expression, we hypothesized that modification of post-translational signaling reflected by changes in phosphorylation events could be responsible. To further characterize these mechanisms, we analyzed the effects of SB202190 on phosphorylations involved in key intracellular signaling cascades, such as the regulatory phosphorylations of ERK-, JNK-, and PKB/Akt as well as S6-phosphorylation, and compared these effects with those of BIRB-796 in HT29 cells. While BIRB-796 did not affect the basal phosphorylation of these components, SB202190 strongly suppressed the phosphorylation of ERK1/2-T202/Y204 and S6 ribosomal protein-S235/236, significantly inhibited the PKB/Akt-T308 phosphorylation and up-regulated Phospho-JNK1/2-T183/Y185 (Figure 7A).

We then asked the question, whether small molecules, which suppress phosphorylation of ERK1/2 and PKB/Akt similar to SB202190 in HT29 cells, such as PD098059, a specific inhibitor of the ERK upstream kinases MEK1/2, and wortmannin, a specific inhibitor of PI3K, are able to induce vacuolization in these cells. Furthermore, we analyzed whether SB202190-induced JNK activation in HT29 cells is required for SB202190-induced vacuolization. Inhibition of ERK1/2 signaling in HT29 cells by PD098059 did not induce vacuoles (Figure 7B). Similarly, JNK1/2 inhibition had no effect on SB202190- induced vacuole formation (Figure 7B), indicating that modulation of ERK1/2 and JNK1/2 activity by SB202190 is not responsible for vacuolization in HT29 cells. Interestingly, treatment of HT29 cells with the PI3K inhibitor wortmannin induced transient vacuolization in HT29 cells, which peaks after about 2 h but disappears almost completely after 4 h (Figure 7C). This indicates that the PI3Kpathway is involved in early vacuole formation in HT29 cells, and that a cross-inhibition of this pathway by SB202190, as suggested by the reduced PKB/Akt phosphorylation, could be the mechanism for SB202190-induced vacuolization. Since the PDK1 inhibitor BX912 is not able to induce vacuolization in HT29 cells (data not shown), the interference of SB202190 with the PI3K-pathway is probably more upstream. When controlling the efficiency of inhibition of ERK1/2 phosphorylation by PD098059, a very effective and specific inhibitor of MEK1/2, for the above experiments, we were excited to see that SB202190 is able to inhibit ERK1/2 phosphorylation in HT29 cells similarly and even in lower concentrations (Figure 7D). Since SB202190 was so far not described as an efficient MEK1/2 inhibitor, we tested various other cell lines for SB202190-mediated inhibition of ERK1/2 phosphorylation (Table 2). Interestingly, only two other human melanoma cell lines, WM793 and WM1617, display an influence of SB202190 on phospho-ERK1/2 (Figure 7D). Since it is known that HT29, WM793 and WM1617 carry the oncogenic BRAF-V600E mutation (30) and the other cells tested do not have this mutation, the cell type-specific effect of SB202190 on ERK1/2 phosphorylation could be BRAF-V600E-dependent. However, regardless to the interesting ability of SB202190 to target the ERK pathway in these cells, this property does not correlate with vacuolization (cf. Table 2).

**Table 2:** Vacuole formation does not correlate with pERK1/2 down regulation.

No	Cell line	Vacuoles	pERK1/2 ↓
1	**AGS**	**+**	**−**
2	**BHK21**	**+**	**−**
3	**Caco-2-Bbe**	**+**	**−**
4	**HEK293T**	**−**	**−**
5	**HeLa**	**−**	**−**
6	**HT29**	**+**	**+**
7	**L929**	**+**	**−**
8	**Sh-SY5Y**	**−**	**−**
9	**WM1617**	−	**+**
10	**WM793**	−	**+**

pERK1/2 ↓ denotes SB202190 induced down regulation of ERK1/2 phosphorylation.

## Discussion

Several studies have shown an evolutionarily conserved role for p38 MAPK in the regulation of autophagy [17], [18], [29]. However, the cell type-specific induction of defective autophagic vacuoles by SB202190 and SB203580 seen in colon cancer HT29 cells, which we extended to other transformed and non-transformed cells types including primary cells, is a p38 MAPK-independent effect of these compounds. This effect of SB compounds in HT29 cells seems to be biphasic, involving an initial phase which leads to initiation of LC3 conjugation and active autophagic degradation followed by the blockade of autophagic proteolysis, leading to the accumulation of acidic vacuoles, p62 and LC3-II. Using further inhibitors of p38 MAPK and a SB-resistant mutant of p38 MAPKα, we could show that inhibition of p38 MAPK is not sufficient for this effect and that the effect even does not require suppression of p38 MAPK activity. Since vacuolization is also detected after Act-D treatment, the cell type-specific changes in pro-autophagic gene expression reported in colon cancer [19], [30] and other cells [17] in response to SB202190 treatment seem to be dispensable for the formation of acidic autolysosomes. The kinetics of vacuole formation, gene expression and FHRE reporter activation suggest that changes in gene expression could be a feed-forward control of already induced vacuoles, or a result of blockade of basal autophagy, leading to accumulation of enlarged autophagolysosomes and cell death. Contradictory data exist in the literature about the correlation between p38 activity, Foxo3A transcriptional activation and pro-autophagic gene expression [19], [31]. Since these studies utilized SB compounds to study the involvement of p38, these effects needs to be re-evaluated.

A wide panel of SB202190 targets has been identified *in vitro* and *in vivo* [6], [7], [32], [33], [34], [35]. Many of these targets including BRAF [36], RIP3 [37], GAK [38], PDK1 [39] and GSKβ [40] have been associated with lysosomal functions, autophagy and caspase-independent cell death. A unique off-target responsible for the SB202190-induced autophagic response seems unlikely, since treatment with a panel of specific and broad spectrum small molecule inhibitors including those targeting previously identified off-targets did not yield the autophagic phenotype in HT29 cells (data not shown). siRNA mediated down-regulation of GAK, GSK3β, RIP2 and RIP3 also did not induce formation of vacuoles (data not shown). It is rather likely that multiple off-target effects culminate in the autophagolysosome formation in a cell type-specific manner. Interestingly, wortmannin induced a transient formation of vacuoles in HT29 cells. The induction of acidic vacuoles by wortmannin has also been reported previously in a normal rat kidney cell line [41]. PI3 kinase family members are shown to positively and negatively regulate autophagy. SB202190-induced autophagic response seems to be biphasic and it could be possible that the initial phase requires PI3 kinase inhibition. Since the PDK1 inhibitor BX912 does not induce vacuolization, only a direct effect of SB202190 on PI3K in a cell type-specific manner could explain the early effects observed. A very recent study by Szyniarowski *et al* have demonstrated the formation of defective autophagosomes upon specific knock down of WNK2 [25]. It is interesting to note that WNK2 amino acid sequence analysis in the p38α-T106 homologous region suggest that this kinase could be SB202190-sensitive (Figure S4). However, as discussed above, multiple off-target effects of SB202190 could probably culminate in non-productive autophagy and accumulation of defective autolysosomes and further detailed studies are required to characterize the mechanism.

Swollen macro-autophagosomes, similar to SB202190-induced vacuoles, were reported recently in primary smooth muscle cells in response to sodium channel blockers [42]. ATP-dependent ion channels and nutrient transporters could be potential non-kinase targets of the SB compounds: SB203580 and SB202190 were shown to affect cellular nucleoside uptake, but this effect was also caused by the inactive variant SB202474 [41] which did not induce vacuoles in HT29 cells. SB202190-induced vacuole formation as well as LC3 conjugation in HT29 cells was enhanced in glucose-pyruvate free medium, but suppressed upon amino acid starvation (M.B.M, unpublished). Targeting cancer cell-specific metabolism for treatment is a sought-after option [43] and the role played by glucose and amino acids for autophagy in HT29 cells and colon cancer should be evaluated in more detail.

Here, we also describe an inhibitory effect of SB202190 on ERK1/2 activation. SB203580 was previously shown to inhibit BRAF and CRAF *in vitro* (31). With the supporting data that SB203580 and SB202190 bind to BRAF and displayed greater affinity for BRAF-V600E *in-vitro* (32), we propose that BRAF-V600E could sensitize the ERK pathway to SB202190. We demonstrate that SB202190 behaves like a BRAF-V600E-specific inhibitor similar to SB-590885 [44] and PLX4720 [45]. BRAF-V600E-mutated cell lines are solely dependent on RAF/MEK/ERK signaling for survival and this oncogene addiction makes RAF/MEK inhibitors relevant compounds for treatment of cancer [46]. Recent studies could show a positive regulatory role for BRAF-V600E in melanoma cell autophagy [36] and enhanced LC3 lipid conjugation in the presence of hyper activated BRAF. However, we demonstrate that the cell line-specific existence of the BRAF-mutation and the ability of SB202190 to inhibit the ERK1/2 pathway do not correlate with the cell line-specific vacuolization induced by SB202190 indicating that other pathways, such as PI3K/PKB/Akt, are responsible for the effects observed.

## Materials and Methods

### Materials

SB203580 (Calbiochem, 559389), SB202474 (Calbiochem, 559387), PD098059 (Calbiochem, 513000), BIRB-796 (Axon Medchem, 1358) and SB202190 (Axon Medchem, 1364) stocks were prepared in DMSO. Phospho specific antibodies against ERK1/2 (#9106), S6 protein (#4858), MK2-T334 (#3041), JNK1/2 (#9255), Akt-T308 (#4056) and total antibodies for LC3b (#2775), Beclin-1 (#3738), p38 (#9212) and ATG12 (#2010) were from Cell signaling Technology. ERK2 (sc-1647) and Hsp27 (sc-1048) antibodies were from Santa Cruz Biotechnology, Other antibodies used were pHsp27-S82 (Invitrogen, 44536), Hsc70 (Stressgen, SPA815), LAMP2 (DSHB-University of Iowa, H4B4-s), pS13-keratin-20 (Gift from Omary, MB) and gapdh (Chemicon international, MAB374). siGENOME SMART pool siRNA for hMAPK14 (p38α) and negative control siRNA were from Dharmacon. Anisomycin, actinomycin-D, acridine orange, neutral red, crystal violet and G418 were purchased from Sigma-Aldrich.

### Cell culture and treatment

HT29 [10], Caco-2 Bbe [47], AGS [48], WM1617 and WM793 [49] cells were maintained in DMEM/F12 medium with 10% FCS and with 20% FCS for Sh-SY5Y [50] (neuroblastoma cells). All other cell lines were grown in DMEM with 10% FCS. Cells were seeded in 12 well plates for experiments and treated as mentioned in the figure legends. For nutrient starvation, cells were washed with PBS and cultured in EBSS (Earle's balanced salt solution). After treatments the cells were washed with PBS and lysed directly in SDS gel loading buffer. LC3-YFP plasmid was transfected into HT29 cells using lipofectamine LTX reagent using manufacturer's protocol. siRNA transfections were performed using Hiperfect reagent (Qiagen) using standard protocol.

### Cell staining and Microscopy

Cells were imaged using a Leica DM IL LED microscope and Leica EC3 camera. Phase contrast or fluorescent images of live cells were taken or cells were fixed with 95% ethanol and stained with 0.5% crystal violet and observed. For AO staining, stock solution (5 mg/mL in water) was added to culture medium to a final concentration of 5 µg/mL, incubated for 15 minutes, medium changed and observed under the microscope with GFP filter settings. For neutral red staining a 1000× stock in DMSO (28 mg/mL) was used, cells were treated similarly and bright field images were acquired. Immunofluorescence staining of HT29 cells was performed as described previously [28].

### FACS-scan for acidic vacuole quantification

Acidic vacuoles were quantified by FACS using a modification of a reported method [51]. Cells after indicated treatments were trypsinized and re-suspended in PBS with 0.5 µg/mL AO and incubated at room temperature for 20 minutes. The suspension was diluted 1∶1 with PBS and the FL3 channel fluorescence analyzed using an Accuri –C6 flow cytometer.

### Immuno blotting

Cell lysates were separated on 7.5–16% gradient denaturing poly acrylamide gels and transferred to Hybond ECL membranes, blocked in 5% BSA-PBS-0.1% Tween-20 for an hour, followed by primary antibodies in the block buffer over night at 4°C. Horse-radish peroxidase-conjugated secondary antibodies (Santa Cruz, sc-2005, 2004, 2033 and 2032) were used and the blots were developed with a self-made ECL detection kit, and the digital chemiluminescence images were taken by a Luminescent Image Analyzer LAS-3000 (Fujifilm).

### Real Time PCR

Cells were treated in 12 well plates and processed as described earlier using SYBR Green chemistry [52]. Actin mRNA was used for normalization. The primer sequences for GABARAP, MAP1LC3A (LC3), BNIP3L and GLUT1 (SLC2A1) are available on request.

### Generation of stable reporter cell line and luciferase assays

Fork Head Response element reporter plasmid was a gift from Dr. Alex Toker and has been reported earlier [53]. The reporter plasmid was co transfected with pEGFP-C1 vector (Clontech) into HT29 cells using Polyethylenimine reagent [54]. 24 hour post transfection cells were treated with 750 µg/mL G418 (geniticin) for additional 10 days. Clones were isolated by serial dilution and cells were analyzed for luciferase activity and GFP fluorescence. For reporter assays, cells were treated in 12 well plates and luciferase activity determined as reported earlier [52]. The firefly luciferase values were normalized to protein content.

### Generation of HT29 cells stably transduced with p38 T106M

Retroviral vector expressing wild-type human p38α is reporter earlier [52]. Thr106 residue was mutated to methionine using Quik Change site directed mutagenesis kit (Stratagene). Virus particles were obtained by transfecting the constructs into 293-GPG cells. HT29 cells were treated with viral supernatants and transduced cells selected in the presence of 600 µg/mL Hygromycin-B.

## Supporting Information

Figure S1 **Wortmannin (Wm) and Chloroquine (CQ) inhibit SB202190 induced vacuole formation.** HT29 cells in complete growth medium were treated with 5 µM SB202190 alone or in combination with with 1 µM Wm for 24 hours (**A**), 70 µM CQ for 6 hours (**B**).
(TIF)

Figure S2 **Nutrient starvation induced autophagic proteolysis is partially inhibited by SB202190.** (**A**) HT29 cells were left in complete growth medium or were nutrient starved in EBSS (Earle's balanced salt solution) alone or in combination with SB202190 or Leupeptin for indicated time points and samples were analyzed by western blotting with the indicated antibodies. The band intensities for LC3-II (**B**) and p62 (**C**) were quantified by image J software, normalized to gapdh and plotted.
(TIF)

Figure S3 **p38α knock down does not induce significant vacuolation in HT29 cells.** HT29 cells were transfected with the indicated siRNAs or were left untreated. Images were acquired 48 h post transfection (**A**) and the percentage of vacuolated cells, calculated from three independent images were plotted with standard deviation (**B**). Cells were lysed and analyzed by western blotting for p38α expression. The percentage expression is plotted after normalization to loading control.
(TIF)

Figure S4 **WNK2 (“with no lysine kinase 2”) is a potential SB target.** Alignment of human p38α (iso-2) and WNK2 (human) kinase domains. Red box indicates the conserved lysine (K) residue in subdomain II which is replaced by cysteine (C) in WNK2. The blue box indicates the gate keeper residue, T106 in p38α which is M/Q/L (bulky residues) in most kinases, which determines the sensitivity to the SB compounds.
(TIF)
