# Supplementary figures and images for: SB202190-Induced Cell Type-Specific Vacuole Formation and Defective Autophagy Do Not Depend on p38 MAP Kinase Inhibition
Source: PLoS One. 2011 Aug 10;6(8):e23054. doi: 10.1371/journal.pone.0023054 (PMC3154272; doi:10.1371/journal.pone.0023054)

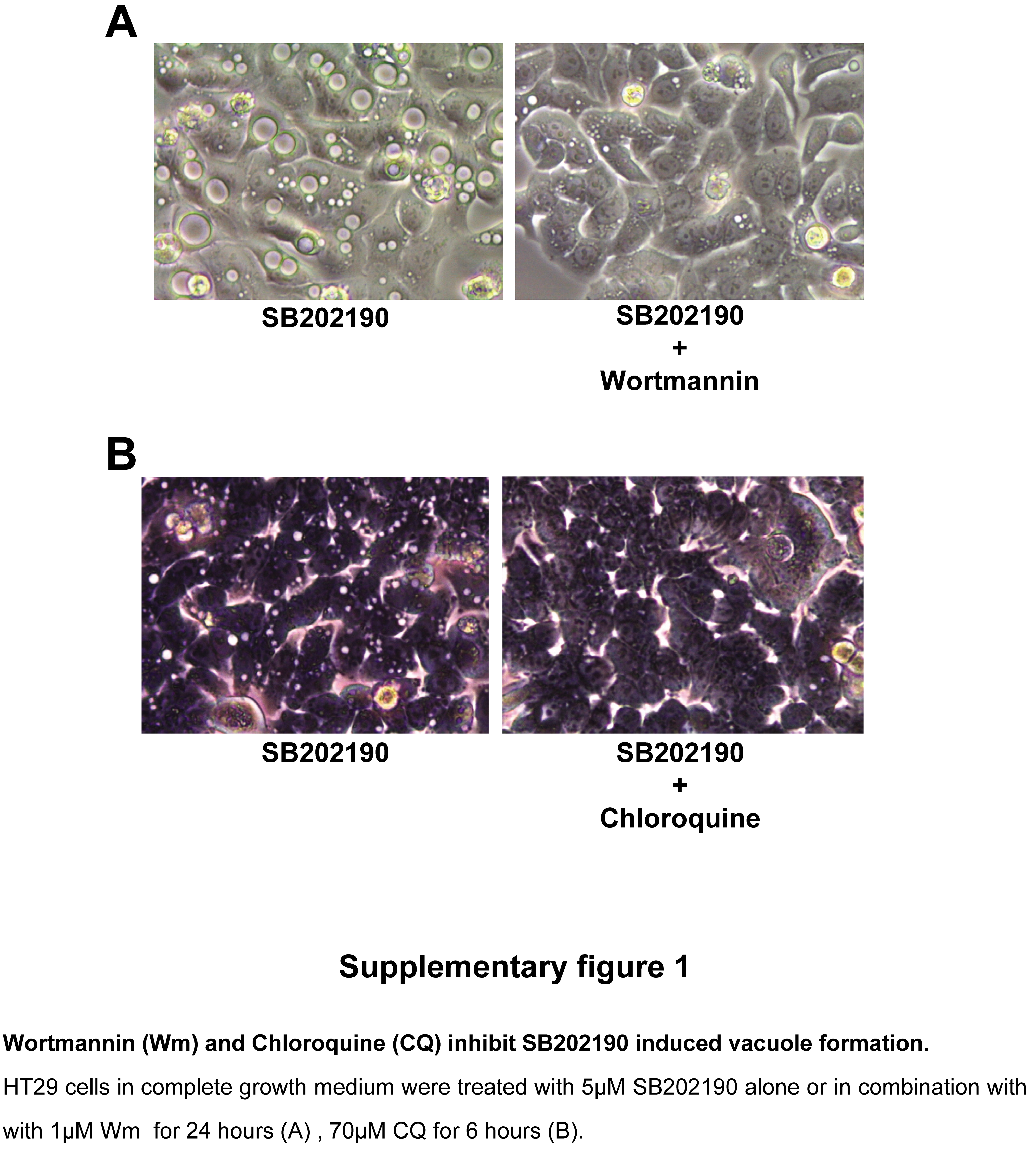

Supplement: Figure S1 — Wortmannin (Wm) and Chloroquine (CQ) inhibit SB202190 induced vacuole formation. HT29 cells in complete growth medium were treated with 5 µM SB202190 alone or in combination with with 1 µM Wm for 24 hours (A), 70 µM CQ for 6 hours (B). (TIF) [file pone.0023054.s001.tif]

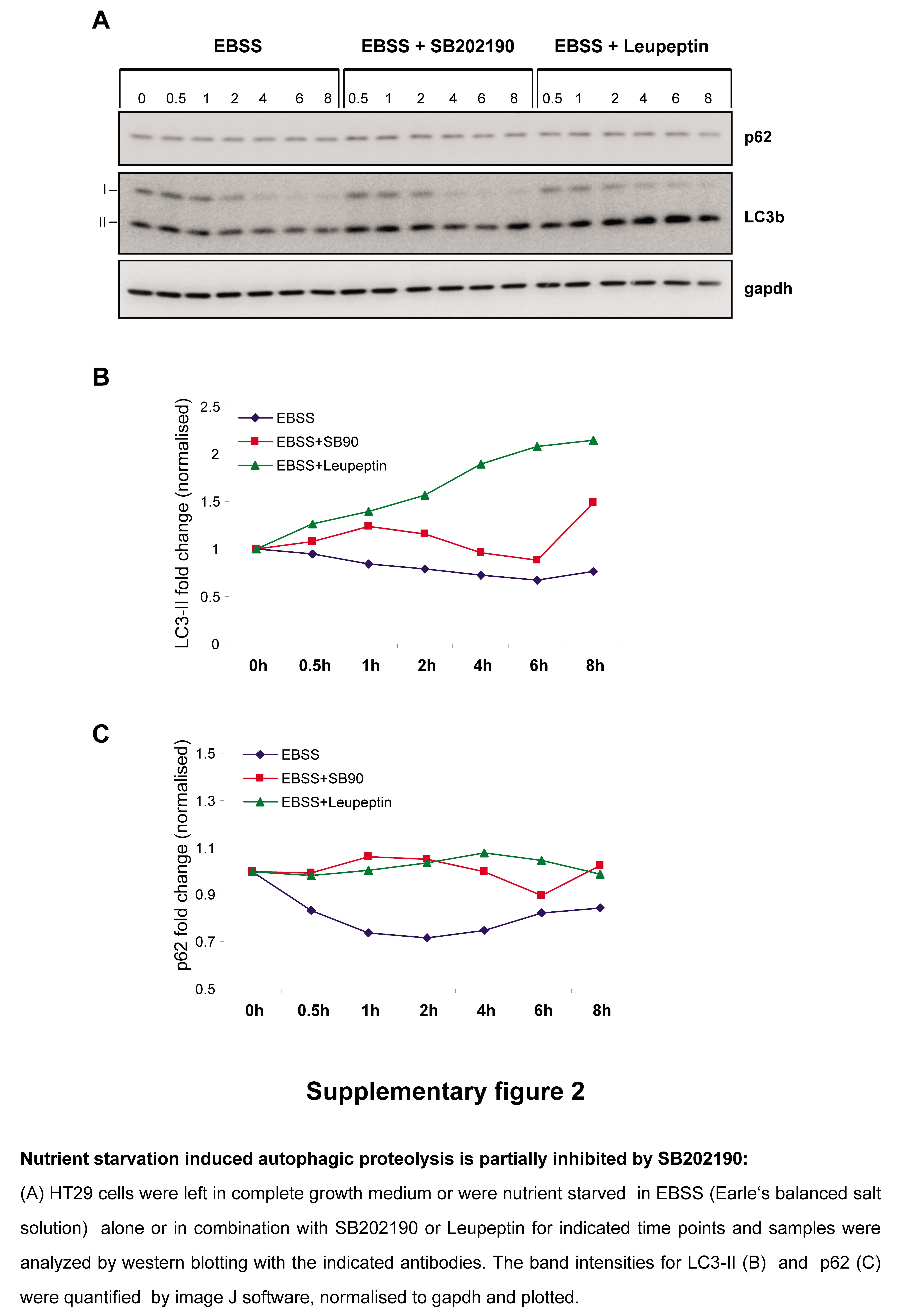

Supplement: Figure S2 — Nutrient starvation induced autophagic proteolysis is partially inhibited by SB202190. (A) HT29 cells were left in complete growth medium or were nutrient starved in EBSS (Earle's balanced salt solution) alone or in combination with SB202190 or Leupeptin for indicated time points and samples were analyzed by western blotting with the indicated antibodies. The band intensities for LC3-II (B) and p62 (C) were quantified by image J software, normalized to gapdh and plotted. (TIF) [file pone.0023054.s002.tif]

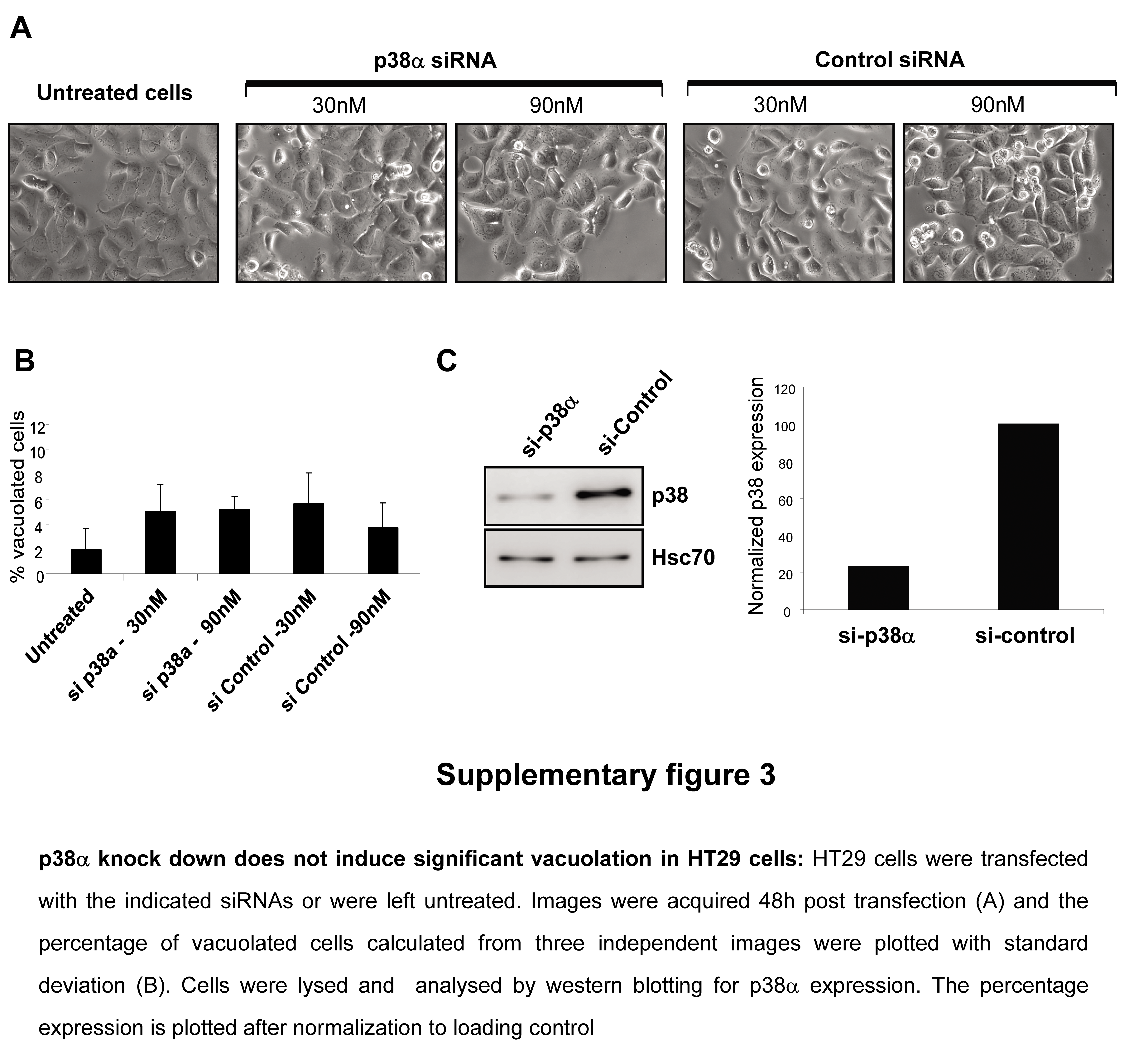

Supplement: Figure S3 — p38α knock down does not induce significant vacuolation in HT29 cells. HT29 cells were transfected with the indicated siRNAs or were left untreated. Images were acquired 48 h post transfection (A) and the percentage of vacuolated cells, calculated from three independent images were plotted with standard deviation (B). Cells were lysed and analyzed by western blotting for p38α expression. The percentage expression is plotted after normalization to loading control. (TIF) [file pone.0023054.s003.tif]

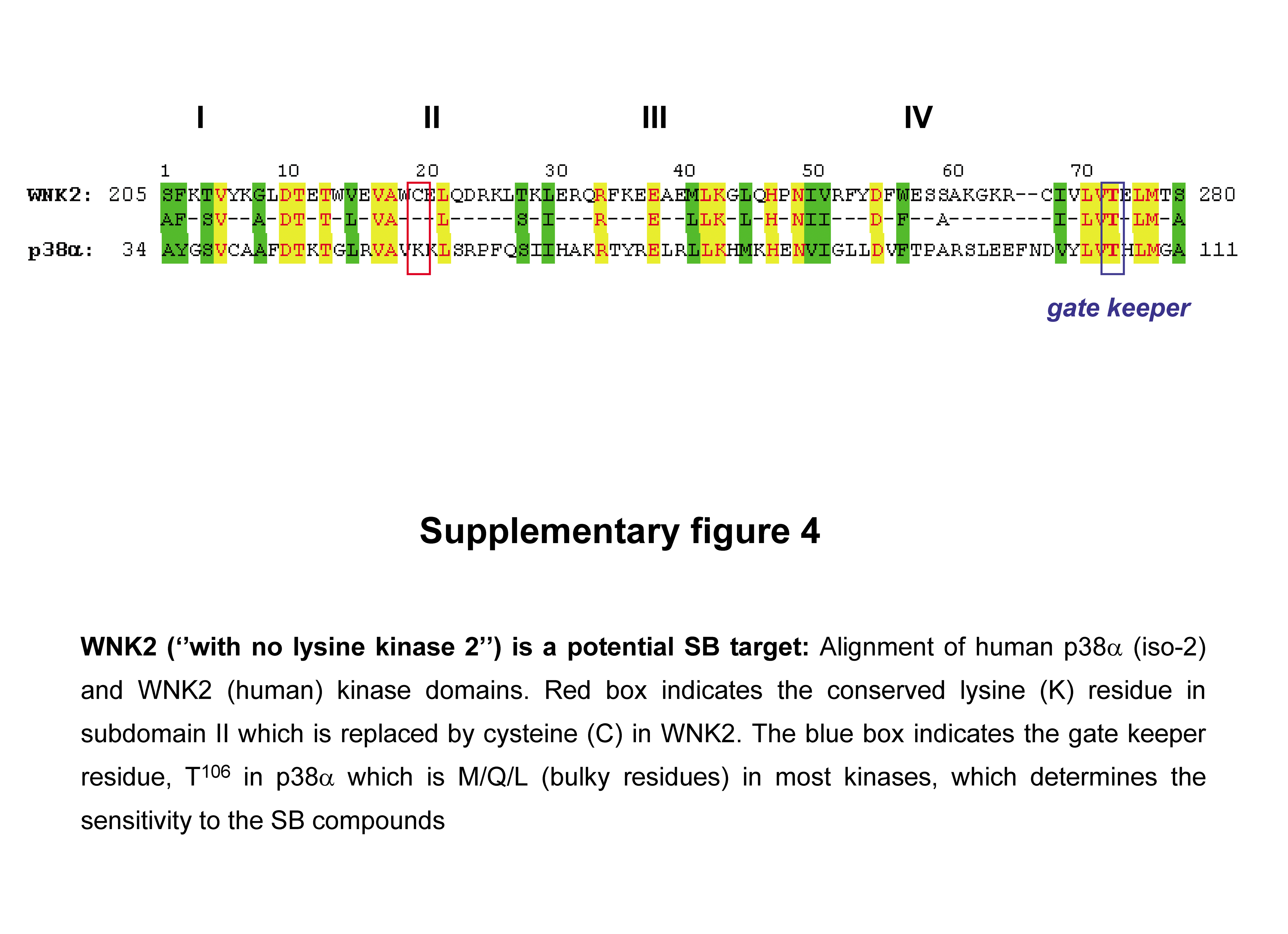

Supplement: Figure S4 — WNK2 (“with no lysine kinase 2”) is a potential SB target. Alignment of human p38α (iso-2) and WNK2 (human) kinase domains. Red box indicates the conserved lysine (K) residue in subdomain II which is replaced by cysteine (C) in WNK2. The blue box indicates the gate keeper residue, T106 in p38α which is M/Q/L (bulky residues) in most kinases, which determines the sensitivity to the SB compounds. (TIF) [file pone.0023054.s004.tif]
